# Feasibility Study to Assess the Impact of a Lifestyle Intervention during Colorectal Cancer Screening in France

**DOI:** 10.3390/nu13113685

**Published:** 2021-10-20

**Authors:** Inge Huybrechts, Nathalie Kliemann, Olivia Perol, Anne Cattey-Javouhey, Nicolas Benech, Aurelia Maire, Tracy Lignini, Julien Carretier, Jean-Christophe Saurin, Beatrice Fervers, Marc J. Gunter

**Affiliations:** 1 International Agency for Research on Cancer, CEDEX 08, 69372 Lyon, France; nathalie.kliemann@gmail.com (N.K.); LigniniT@iarc.fr (T.L.); gunterm@iarc.fr (M.J.G.); 2 Centre Léon Bérard, 69008 Lyon, France; olivia.perol@lyon.unicancer.fr (O.P.); Anne.CATTEY-JAVOUHEY@lyon.unicancer.fr (A.C.-J.); Aurelia.MAIRE@lyon.unicancer.fr (A.M.); julien.carretier@lyon.unicancer.fr (J.C.); beatrice.fervers@lyon.unicancer.fr (B.F.); 3 Service d’Hépato-Gastroentérologie, Hôpital Edouard Herriot, Hospices Civils de Lyon, 69003 Lyon, France; nicolas.benech@chu-lyon.fr (N.B.); jean-christophe.saurin@chu-lyon.fr (J.-C.S.)

**Keywords:** feasibility, lifestyle intervention, colorectal cancer screening, hospital setting, France

## Abstract

Current evidence suggests that 30–50% of cancers are attributable to established lifestyle risk factors. Cancer-screening has been identified as an opportunity for delivering advice on lifestyle behaviour change for cancer prevention. This study aimed to evaluate the feasibility and acceptance of promoting advice on the latest evidence-based lifestyle recommendations for cancer prevention at the time of colorectal cancer screening at two hospitals in Lyon, France. This feasibility study included 49 patients (20 men and 29 women) who were invited for colonoscopy. Patients received a leaflet with lifestyle recommendations for cancer prevention, accompanied with a logbook to plan and monitor their behavioural changes. Feedback from patients, hospital staff, and researchers was received via evaluation questionnaires (*n* = 26) completed after testing the educational material for at least two weeks and via two focus group discussions (*n* = 7 and *n* = 9 respectively) organized at the end of the study. All interviewed patients were interested in lowering their cancer risk, and the majority felt ready to change their lifestyle (88%), although most did not know how to decrease their risk of cancer (61%). All patients found the educational material easy to understand and sufficiently attractive and 50% of the patients reported having achieved at least one of the healthy behaviours recommended within the two weeks following the intervention. All hospital staff and almost all patients (92%) involved found that the screening program and the visits planned for colonoscopy was an appropriate moment to provide them with the educational material. This feasibility study has shown that the content, paper-based format, and time of delivery of the intervention were adequate. Health professionals seem to be willing to provide lifestyle recommendations, and patients appear interested in receiving advice for lowering their cancer risk during screening visits.

## 1. Introduction

Colorectal cancer (CRC) is the fourth most commonly diagnosed malignancy and the third leading cause of cancer death in the world, accounting for around 1.9 million new cases and almost 935,000 deaths in 2020 [1]. Given current demographic projections, the global burden of CRC is anticipated to increase by 60% to over 3 million new cases and 1.6 million cancer deaths annually by 2040 [2]. The incidence of CRC varies widely across geographic regions with the highest incidence in higher income countries [3]. In France, for example, CRC is the second most common cancer and the second leading cause of cancer death, accounting for more than 48,061 new cases and 20,953 deaths in 2020 [1].

CRC is a complex disease with a number of recognised risk factors. Advancing age, male sex, family history of CRC, inflammatory bowel disease, smoking, excessive alcohol drinking, overweight and obesity, low levels of physical activity and sedentary lifestyle, diabetes and high consumption of red and processed meat are established risk factors [4,5,6,7]. Decades of research have specifically focused on dietary factors: some studies have suggested a protective effect of diets rich in fruit, vegetables, fish, fibre and whole grains, calcium and dairy products against colorectal cancer [5]. Overall, it has been estimated that lifestyle factors could account for up to 40% of CRC cases worldwide [8]. Recent estimates indicate that in France, 56% of CRC cases in men and 74% of CRC cases in women are attributable to modifiable risk factors. Thus, up to 19,000 CRC cases per year could be prevented in France by improving unhealthy lifestyle behaviours [9].

The World Cancer Research Fund’s (WCRF) Continuous Update Project (CUP) is the world’s largest source of scientific research on cancer prevention and survivorship through diet and physical activity. The CUP analyses global research on how diet and other lifestyle factors are associated with the risk of developing CRC [10], to provide a basis for lifestyle recommendations. More recently, in 2018, the WCRF updated its latest lifestyle recommendations for cancer prevention [10], providing the most up-to-date evidence-based lifestyle recommendations for preventing CRC as well as other cancers. Recent longitudinal studies have demonstrated that adherence to the WCRF recommendations for cancer prevention are associated with 5–17% reductions in CRC incidence and 10–13% reductions in CRC mortality [11,12,13,14,15]. Therefore, developing effective and sustainable interventions promoting lifestyle changes for CRC prevention is of high public health interest. Cancer-screening has been identified as an important milestone that could provide an ideal opportunity for delivering advice on behaviour change for cancer prevention [16]. Berstad and colleagues recently highlighted the importance of including lifestyle counselling as a part of CRC screening since individuals with a positive screening result may be inclined to a less healthy lifestyle compared with those who had a negative screening result [17].

Screening attendance represents a time-window whereby patients may be more receptive to advice on lifestyle change, which has been described as a ‘teachable moment’ [16]. Screening attendance may influence an individual’s perception of their personal risk for CRC, which in turn may prompt motivation to change behaviour. Indeed, a recent study showed that adults eligible for cancer screening and who were not adhering to guidelines were willing to receive lifestyle advice during screening, regardless of its results [18]. While there is little evidence to support the idea that screening prompts spontaneous behaviour change [19], interventions delivered at CRC and mammography screening in the U.K have shown promising results, such as weight loss and increased physical activity [20,21]. However, these interventions were very intensive and there is still a need to test the effect of brief interventions that promote long-term behaviour change as part of screening programs to allow widespread implementation. A recent brief habit-based weight loss intervention promoting a set of everyday healthy eating and physical activity behaviours associated with cancer prevention in patients with obesity from primary care in the UK has shown promising results, such as the maintenance of a significant weight loss over 24 months [22]. Since habit formation advice is simple, easily scalable, and a recommended approach to be used with patients [23], it may be a feasible approach to deliver evidence-based lifestyle recommendations in the cancer screening context.

The CRC screening program in France may be an ideal setting for delivering advice on lifestyle behaviours. This is a population-based nationwide cancer screening program which has been rolling out since 2009. In 2015, the immunochemical test (FIT) replaced the conventional guaiac faecal occult blood test (gFOBT), improving the participation rate [24,25]. The programme targets those aged 50 to 74 years old, who often have other lifestyle related comorbidities, such as obesity, type 2 diabetes mellitus, and cardiovascular disease. Therefore, promoting healthy lifestyle advice to the screened population has the potential to reduce the risk of cancer as well as other related comorbidities and improve prognosis and quality of life. When developing and testing a lifestyle intervention for CRC prevention, it would be the most efficient to first target higher risk population groups before expanding the intervention to the entire screening population. High risk patients include those with positive FIT, a family history of CRC, or patients who score positive (≥5) for a validated score consisting of simple clinical factors that successfully estimates the likelihood of detecting advanced colorectal neoplasia in asymptomatic Caucasian patients [26].

Despite this, there is still little evidence on the impact of promoting the evidence-based lifestyle recommendations during CRC screening among individuals at higher risk. There is also a need to extend our efforts to gain understanding of the potential pathways that can explain how lifestyle factors may prevent CRC development [27]. Therefore, the LIFE-SCREEN intervention trial aims to investigate the hypothesis that advice on lifestyle recommendations for cancer prevention at CRC screening among individuals classified as higher risk will promote greater adherence to cancer prevention recommendations, as well as improve the quality of life, biomarkers of cancer risk, physical fitness, and body weight. The current feasibility study presented in this manuscript aimed to evaluate the feasibility and acceptance of the LIFE-SCREEN intervention. Specific objectives were to obtain information on (i) participants’ and health professionals’ feedback on the educational material; (ii) health professionals’ willingness to recruit participants, and (iii) participants’ awareness of the risk factors for CRC and willingness to change their lifestyles. In addition, we aimed to conduct focus group meetings with patients, health professionals, and experts in the field in order to explore their feedback on the intervention content, format and delivery in great detail.

## 2. Materials and Methods

### 2.1. Study Design and Setting

This study was a single arm, two-center feasibility study of the LIFE-SCREEN intervention that aims to provide advice on evidence-based lifestyle recommendations for cancer prevention at CRC screening among individuals at higher risk of CRC. The feasibility study was carried out in 2019 at two hospitals in Lyon, France, L’hôpital Edouard Herriot (HEH) and Centre Léon Bérard (CLB). The LIFE-SCREEN intervention is registered with clinicaltrials.gov (Ref ClinicalTrials.gov Record PP201907-26) where more details on the feasibility study design and methods can be found. The study was conducted according to the guidelines of the Declaration of Helsinki, and approved by the Institutional Review Board (or Ethics Committee) of International Agency for Re-search on Cancer (IEC Project No. 19-26; reviewed and approved on 3 February 2020).

### 2.2. Participants

Considering the feasibility study objectives we aimed to recruit at least 30 participants [28]. Eligible patients were adults >18 years without previous cancer diagnosis, who were capable to provide informed consent, and that were attending for colonoscopy as part of CRC screening.

### 2.3. Lifestyle Intervention

The LIFE-SCREEN intervention aimed to deliver lifestyle advice based upon the most recent recommendations for cancer prevention published by the World Cancer Research Fund (WCRF) [8]. These recommendations entail the following target behaviours: (1) be a healthy weight; (2) move more; (3) enjoy more grains, vegetables, fruits, fish, and dairy products; (4) avoid high-calorie foods rich in fat, salt, and sugar; (5) limit consumption of red and processed meat; (6) limit alcoholic drinks; (7) limit consumption of sugar-sweetened drinks; (8) do not rely on supplements. The behavioural approaches were informed by relevant behaviour change theories and models, such as the Teachable Moments Heuristic model and the Habit formation theory. The concept of Teachable Moments (TMs), that is, naturally occurring life or health events that may prompt risk-reducing health behaviours [16], has been considered a strong foundation for widely-accepted health behaviour models. In addition, the intervention was based on the habit formation theory, in order to promote lasting healthy lifestyle behaviours. Habit-based interventions promote the repetition of target behaviours in a consistent context in order to make them become more automatic and habitual [29,30,31]. They also promote self-regulatory skills (e.g., goal-setting, planning, self-monitoring, and feedback on performance) in order to translate the intended behaviour into action and override unwanted automated responses [29,32].

The intervention was delivered as a self-coaching leaflet and booklet materials containing the advice on lifestyle recommendations together with strategies on how to achieve and maintain these behaviours. The material also contained instructions on how to set up specific goals (e.g., including what, how, where, and when) to achieve the target behaviours and repeat them at the same time and in the same place in order to improve the patient’s likelihood of forming habits and maintaining their behaviour changes. It also provided the participants with instructions on how to track their progress (e.g., using printed booklets to monitor their behaviour), and to amend their plans when these seem inefficient in reaching their target goals.

Health professionals introduced the intervention to eligible patients during pre-colonoscopy (HEH) or post-colonoscopy visit (CLB), depending on the hospital and provided the informed consent letter. During the consultation, health professionals briefly endorsed the importance of the intervention for helping them to achieve and maintain healthy lifestyles and in turn to reduce their colorectal cancer risk. Interested patients, who signed the informed consent for this feasibility study, were provided with the educational material and were instructed to follow the intervention for at least 2 weeks before completing the feasibility evaluation questionnaire.

### 2.4. Measurements

At baseline, participants were required to answer questions on socio-demographics, reason for the colonoscopy and the type of appointment (pre- or post-colonoscopy), their interest and knowledge on how to reduce colorectal cancer risk, readiness to change their behaviours and interest in following the intervention. At the end of the two-week intervention, participants were invited to complete a feasibility evaluation questionnaire containing closed and open questions to obtain their feedback on the educational material, format, and delivery as well as questions on compliance to the intervention over 15 days. Participants were asked to post their completed evaluation questionnaire back using the pre-paid return envelope or to bring it with them during the focus group meetings. It should be noted that no objective measures were used to control the patient’s behaviours in this feasibility study; all measures were self-reported by the patients. Health professionals were also requested to complete an evaluation questionnaire on the intervention delivery and content.

In addition, two focus group discussions were conducted at the end of the feasibility study to discuss in depth the feedback on the intervention content, format, and delivery. One focus group gathered the researchers and (para-)medical staff involved in the feasibility study and the second one invited all the patients who took part in the study. These focus group meetings were logistically organized at one of the hospitals involved in the intervention and moderated by the researcher coordinating the LIFE-SCREEN intervention. Each session began with a PowerPoint presentation that provided an overview of the LIFE-SCREEN study, design, and methods. Next the moderator asked the participants for feedback on the intervention content, format, and delivery. Both focus groups were audio recorded and lasted over one hour each. Participants were offered a free lunch meal to thank them for taking part.

### 2.5. Data Analysis

Descriptive analysis of the study population was performed. No association analysis was undertaken given the small sample, which was not powered to detect significant differences. Two researchers involved in the study independently assessed the audios and minutes from the focus groups and identified the main suggestions and feedback received. They met to discuss aspects such as recruitment and delivery acceptability, barriers for lifestyle intervention, content of the educational material and any other issue that should be considered when conducting the full RCT. A consensus list of improvements was then defined and used to optimize the study protocol further.

## 3. Results

This feasibility study included 49 patients (20 men and 29 women), aged from 23 to 75 years (Table 1). Most of the patients received the invitation and the educational material at their post-colonoscopy visit (84%). All patients who participated in the feasibility study were interested in lowering their CRC risk, although most of them did not know how to decrease their risk (61%). Eighty-eight percent of the respondents felt ready to change their lifestyles with the aim to lower their CRC risk.

At two-week’s follow-up, a total of 26 patients (53%) completed the evaluation questionnaire. Regarding compliance to the intervention, presented in Table 2, half of the patients (50%) reported having achieved at least one target behaviour using the monitoring sheets. Most of them made plans to achieve at least one behaviour (58%) and made amendments to the plans when necessary (58%). Importantly, the majority of patients intended to continue following the intervention after the end of the study (74%).

Table 3 shows patients feedback on the intervention delivery and content collected through evaluation questionnaires. All patients said they easily understood the information included in the educational material and most of them found the material sufficiently attractive, complete and in line with their expectations (88%). Although most patients (92%) found the material covered the kind of information they would expect, some reported having suggestions to improve it (27%). Suggestions were made such as providing more plant-based and vegetarian recipes and practical tips on how to achieve their goals (e.g., examples of activities adapted to an ageing population to maintain their fitness, etc.). Interestingly, 54% of the patients preferred to stick to the non-digital (e.g., paper-pencil) tools to monitor their behaviours rather than using digital tools. Most of them found that the CRC screening program and the visits planned for colonoscopy were right/appropriate moments to provide them with the educational material (92%). Similar feedback was obtained in the patients’ focus group, composed of three patients (two women and one man) involved in the feasibility study.

Feedback from experts and health professionals involved in the study was obtained via focus group discussions and evaluation questionnaires. The focus group was conducted with four health professionals and three experts in the field. They suggested to screen participants for their current adherence to the recommendations for cancer prevention at baseline and exclude those with a high score (complying with the recommendations), as they would not have much room to improve their behaviours. However, they emphasized that the study protocol must ensure that the extra work for the surgeons and other health professionals is kept to the minimum (~5 min/patient) to ensure intervention feasibility. They also underlined the importance of testing participants on knowledge about healthy lifestyles and ‘knowledge change’, as the knowledge of many patients seemed rather low. Regarding the time of the delivery, it was concluded that both pre- and post-colonoscopy are possible. Although it was concluded that both pre- and post-colonoscopy are possible, it was agreed that the most optimal time to deliver the educational material was during the pre-colonoscopy visit, followed by a follow-up intervention (referring to the educational material received at baseline) taking place during the post-colonoscopy visit. Experts and health professionals found that the material could be improved by providing patients with fridge magnets containing messages about the recommendations; recipe books with low-cost vegetarian recipe ideas; and more tips on how to get more active (especially for older patients). They also suggested that the recommendation for quitting smoking should be more prominent.

## 4. Discussion

The importance of promoting lifestyle changes to prevent cancer has been highlighted in the scientific literature as well as in the lay press [33,34]. Although the time at which an individual undergoes cancer screening could be considered as a teachable moment [20,21], so far evidence regarding the effectiveness of evidence-based lifestyle advice administered during cancer screening is still scarce. Nevertheless, the necessity and importance of including lifestyle counselling as a part of CRC screening has been demonstrated in particular among individuals with a positive screening result, as they may be inclined to a less favourable lifestyle compared with those who tested negative at screening [17]. Therefore, this feasibility study was set up to evaluate the feasibility of an intervention study aimed at delivering evidence-based lifestyle advice during CRC screening among individuals at higher CRC risk, as this may have high public health potential.

In summary, this feasibility study showed that the content, paper-based format, and time of delivery of the intervention (both pre- and post-colonoscopy) were adequate and well accepted by the health professionals and patients. 

Previous studies already investigated the impact of lifestyle interventions during cancer screening, though with mixed results [35,36,37,38,39,40]. Although the colorectal cancer screening has already been evaluated as a potential teachable moment for lifestyle interventions [35], the interventions evaluated so far were rather intensive and personalised lifestyle interventions that require important time investments of (para)-medical staff. Here, our results suggest that a simple, well-documented intervention before or after colonoscopy may be sufficient to impact patient lifestyle. Indeed, following our intervention, half of the patients reported having achieved at least one of the healthy behaviours recommended within the two weeks after the first visit. These results should be confirmed in a larger randomised controlled trial.

Patients and health professionals provided valuable suggestions and feedback for further improvement of the study protocol and educational material, leading us to include a “recipe and practical tips book”. The paper-based format of the intervention will be maintained, but patients will also have the option to get access to the electronic (e.g., pdf) version of all documents. Patients will also be allowed to choose whether they wish to receive reminders of the recommendations sent by text-messages and/or emails during the 12-month follow-up. The delivery of the intervention will take place during the pre-colonoscopy visit and a follow-up intervention (referring to the educational material received at baseline) will take place during the post-colonoscopy visit. However, the inclusion in the study will be conditional to the colonoscopy results, as patients who receive CRC diagnosis during the post-colonoscopy visit are not considered eligible for the intervention study that aims at patients at higher risk.

The proposed intervention is expected to promote greater adherence to cancer prevention recommendations and have an effect on quality of life, biomarkers of cancer risk, physical fitness, and body weight among higher CRC risk population groups. The integration of these well-established continuous frameworks, namely the CRC screening program and the WCRF-CUP cancer prevention program that continuously updates the evidence on lifestyle risk factors and cancer risk, ensures the sustainability of this intervention program and the potential for further expansion to other cancer screening programs in France and beyond. If successful, this intervention trial could inspire similar initiatives for other cancer types that have well established cancer screening programs and can be expanded to the full screening population.

The fact that the intervention will be implemented in hospitals, alongside the CRC screening routine procedures, makes the access to blood, faecal, and tissue samples from participants easier and will allow us to explore the effect of the intervention on nutritional, inflammation, metabolic health and microbial biomarkers that could provide important information on the mechanisms of CRC development and prevention targets.

## 5. Future Perspectives

As stated previously, no standardised protocols or recommendations are available for individuals at higher risk of CRC. If this evidence-based lifestyle intervention confirms the hypothesis that adherence to the latest evidence-based lifestyle guidelines improves following such an intervention, which in turn reduces CRC risk and improves the quality of life, then the intervention can be implemented nationwide and beyond. The recommendations will be developed as a visual representation that is interpretable by non-literate patients to consider and guarantee social equality among the target population. In addition, the lifestyle advice given in this intervention is aimed to limit the burden for the hospital staff involved to a minimum so that it can easily replace routine care when upscaling to a nation-wide intervention.

In the future, this intervention protocol could also be tested in primary care by providing the healthy lifestyle advice to all participants involved in CRC screening, regardless of their FIT test result.

In addition to cancer, rates of other non-communicable diseases (NCDs) continue to rise, affecting the poorest and most vulnerable populations. The intervention targets a higher CRC risk population aged 35 or over, who often have other lifestyle related comorbidities, such as obesity, type 2 diabetes mellitus, and cardiovascular disease. These diseases share common risk factors, as for example, altered lipid levels, inflammation and abnormal glucose metabolism [41,42]. Hence, promoting healthy lifestyle advice to this population has the potential to also reduce the risk of other related NCDs. 

Lastly, this intervention will also shed light on changes in biomarkers relevant for cancer prevention, allowing a better understanding of the putative mechanisms involved.

## 6. Conclusions

This feasibility study has shown that the content, paper-based format, and timing of delivery of the intervention (pre- and/or post-colonoscopy) were adequate and well-accepted by both the health professionals and patients. Health professionals seem to be willing to provide lifestyle recommendations and patients seem interested in receiving advice for lowering their cancer risk during screening visits. Considering the interesting finding that more than half of the patients made plans and adjustments to achieve one of the healthy behaviours recommended in the educational material and that half of the patients reported having achieved at least one of the healthy behaviours recommended within two weeks of receiving the intervention, this LIFE-SCREEN intervention is expected to promote greater adherence to cancer prevention recommendations. Therefore, this LIFE-SCREEN intervention will now be evaluated within the context of a funded randomised-controlled trial.

## Figures and Tables

**Table 1:** Baseline information (*n* = 49).

	*n* (%) or Mean (Min-Max)
Sex	
Men	20 (41%)
Women	29 (59%)
Age	56.6 (23–75) *
Hospital visit at which the intervention was performed	
Pre-colonoscopy	4 (8%)
Post-colonoscopy	41 (84%)
Hospitalization for colonoscopy	4 (8%)
Reason for the colonoscopy	
Family history of Colorectal cancer (CRC)	7 (14%)
CRC symptoms	13 (27%)
CRC screening (positive FIT test)	7 (14%)
Medical follow-up (e.g., Lynch)	22 (45%)
Interested in reducing CRC risk	
Yes	49 (100)
No	0
Knowledge on how to reduce CRC risk	
Yes	19 (39%)
No	30 (61%)
Do healthy lifestyle behaviours help reduce CRC risk?	
Yes	41 (84%)
No	2 (4%)
Don’t know	6 (12%)
Ready to change lifestyle behaviours	
Yes	43 (88%)
No	1 (2%)
Don’t know	5 (10%)
Interested in taking part in LIFE-SCREEN	
Yes	45 (92%)
No	2 (4%)
Do not know	2 (4%)

* Mean and median age were equal (57 years).

**Table 2:** Follow-up information on compliance to the intervention over 15 days (*n* = 26).

Compliance to the Intervention	*n* (%)
Achieved at least one healthy behaviour using the tick sheets	
Yes	13 (50%)
No	10 (38%)
Missing	3 (12%)
Made plan(s) to achieve at least one healthy behaviour	
Yes	15 (58%)
No	9 (34%)
Missing	2 (8%)
Made adjustment to plan(s) to achieve at least one healthy behaviour	
Yes	14 (58%)
No	9 (34%)
Missing	3 (8%)
Intends to continue following the intervention?	
Yes	19 (74%)
No	7 (26%)
Missing	-

**Table 3:** Follow up information on intervention delivery and content collected via evaluation questionnaires (*n* = 26).

Feedback on the Intervention Delivery and Content	*n* (%)
Interested in taking part in this intervention if attending cancer screening again in the future	
Not very interested	6 (23%)
Somewhat interested	11 (42%)
Very interested	5 (20%)
Not applicable or missing	4 (15%)
Interested in taking part in this intervention even if randomied to intervention or control condition	
Not very interested	5 (20%)
Somewhat interested	12 (46%)
Very interested	5 (19%)
Not applicable or missing	4 (15%)
Interested in receiving text-messages and emails reminding about the healthy behaviours	
Not very interested	9 (35%)
Somewhat interested	9 (35%)
Very interested	6 (23%)
Not applicable or missing	2 (7%)
The educational material was easy to understand	
Yes	26 (100%)
No	0
The educational material covered the kind of information you would expect	
Yes	24 (92%)
No	0
Missing	2 (8%)
The educational materials are attractive and eye-catching	
Yes	23 (88%)
No	1 (4%)
Missing	2 (8%)
Is there any other information or tips that you would like to see in the leaflet or logbook?	
Yes	7 (27%)
No	18 (69%)
Missing	1 (4%)
Was the timing of the delivery adequate?	
Yes	24 (92%)
No	1 (4%)
Missing	1 (4%)
Would it be helpful to have access to a mobile or web-based app to monitor your behaviours?	
Yes	11 (42%)
No	14 (54%)
Missing	1 (4%)

## Data Availability

Access to anonymized data and the intervention/educational material can be requested via email through the corresponding author.
